# Clinical characteristics and prognostic analysis of deep venous thrombosis in pediatric convulsive status epilepticus patients: A retrospective study

**DOI:** 10.1097/MD.0000000000045483

**Published:** 2025-10-24

**Authors:** Jie Ren, Chao Tang, Dan Sun

**Affiliations:** a Department of Critical Care Medicine, Wuhan Children’s Hospital, Tongji Medical College, Huazhong University of Science & Technology, Wuhan, China; b Department of Orthopaedic and Maxillofacial Surgery, Wuhan Children’s Hospital, Tongji Medical College, Huazhong University of Science & Technology, Wuhan, China; c Department of Neurology, Wuhan Children’s Hospital, Tongji Medical College, Huazhong University of Science & Technology, Wuhan, China.

**Keywords:** children, convulsive status epilepticus, deep venous thromboembolism, prognosis

## Abstract

This study investigates the clinical characteristics and prognosis of convulsive status epilepticus combined with deep venous thrombosis (CSE-DVT) in children. We retrospectively analyzed the data of CSE-DVT (n = 10) and nonconvulsive DVT (n = 31) admitted to Wuhan Children’s Hospital between January 2014 and May 2023. In the CSE-DVT group, 10 cases (5 males, 5 females) were studied, with a median age of 3.2 years (range 0.2–12.5 years). All of them developed unilateral lower extremity DVT and had markedly elevated levels of serum D-dimers. In the nonconvulsive DVT group, 31 cases (20 males, 11 females) were studied, with a median age of 2.0 years (range 0.1–13.0 years). Fifteen cases (48.3%) developed unilateral lower extremity DVT. Twenty-three cases (74.2%) had markedly elevated levels of serum D-dimers. Compared with the nonconvulsive DVT group, the CSE-DVT group had a larger case number of ventilator support, infection, and intravenous nutrition support (*P* < .05). The CSE-DVT group also had a higher blood platelet count and antithrombin-III, but the activated partial thromboplastin time was shorter than that in the nonconvulsive DVT group (*P* < .05). The mean period of follow-up was 6 ± 2 months. In the CSE-DVT group, none of them had a pulmonary embolism, and 5 cases (50%) had a thrombus-absorbed state. In the nonconvulsive DVT group, 16 patients (51.6%) had a thrombus-absorbed state, and 2 cases (6.4%) had a pulmonary embolism. The deep vein of the lower extremity is the most common site in children diagnosed with CSE-DVT whose D-dimers are markedly elevated. The effective methods to improve prognosis in patients are early diagnosis and timely treatment.

## 1. Introduction

Convulsive status epilepticus (CSE) is one of the common critical illnesses in children, and timely termination of clinical electrical seizures, and accurate identification and diagnosis of underlying causes are particularly important for the prognosis.^[1]^ It has been reported that the CSE short-term fatality rate of children after multidisciplinary standardized diagnosis and treatment is <3%, which is significantly lower than the adult level.^[2]^ However, long-term morbidity and mortality can range from 5% to 17%, depending on how its complications develop.^[3,4]^ Therefore, the connotation of prognosis is not only to save lives but also to cover the prevention and treatment of complications. One of the common complications associated with it is deep vein thrombosis (DVT). As reported in both national and international literature, the incidence of DVT in children is relatively low; however, it has been on an upward trend. Annually, the incidence of DVT in the pediatric population is approximately in the range of 0.14 to 0.21 cases per 10,000 individuals. Notably, this figure climbs to between 0.2% and 0.6% among hospitalized pediatric patients.^[5]^ The primary etiology of DVT in children encompasses the well-known triad: a hypercoagulable state of the blood, injury to the vein walls, and sluggish blood flow.^[6]^ Venous wall damage can be caused by various factors such as surgery, trauma, infection, and the presence of central lines. Meanwhile, conditions like prolonged bed rest, insufficient physical activity, and obesity can contribute to sluggish blood flow.^[1,6]^ Since CSE children often have long-term bed rest, deep venous catheterization, hormone use, etc, they are at risk of hypercoagulability and vascular endothelial damage, which is likely to lead to DVT and affect prognosis.^[7]^ However, the understanding of CSE combined with DVT (CSE-DVT) is not sufficient, and there have been no relevant reports. Meanwhile, other special types of DVT, such as acute lymphoblastic leukemia, which are affected by their primary pathogenesis, cannot represent the clinical characteristics of children with various types of DVT.^[8]^ Based on this, this study analyzed the clinical data of 10 children with CSE-DVT admitted to our center, and compared it with nonconvulsive DVT children, to improve everyone’s understanding of CSE-DVT.

## 2. Methods

### 2.1. Study population

The clinical data of 10 children with CSE-DVT admitted to Wuhan Children’s Hospital, Tongji Medical College from January 2014 to May 2023 were retrospectively analyzed, and 31 nonconvulsive children with DVT admitted during the same period were included as a control group for comparative analysis. To be included in the CSE-DVT group, the following 2 criteria must be met: the seizure has lasted >5 minutes and has not responded to the first-line anti-seizure medication^[2]^; confirmation of new DVT by ultrasound, computed tomography, or magnetic resonance imaging methods. Children who meet the criteria of confirmation of new DVT by ultrasound, computed tomography, or magnetic resonance imaging methods but without CSE are included in the nonconvulsive DVT group. The study was approved by the Medical Ethics Committee of Wuhan Children’s Hospital (2024R152-E01).

### 2.2. Outcomes and data collection

The data collection included the sex, age, underlying diseases, length of stay in the pediatric intensive care unit, duration of invasive ventilation, whether peripherally inserted central catheter was placed, whether infection occurred, whether intravenous nutritional support was provided, whether hormones were used, location of DVT, blood biochemistry, coagulation function, imaging, treatment, and prognosis were collected by the inpatient medical record system. Data collection was independently performed by 2 researchers, with disputed indicators discussed and resolved by a third-party reviewer before final inclusion. Follow-up data were collected by telephone and outpatient medical record system.

### 2.3. Statistical analysis

SPSS version 22.0 statistical software (Armonk) was used to analyze the data. Count data was expressed as frequency and percentage (%), and group comparisons were made by using the *X*^2^ test. The normal distribution of quantitative data was expressed as mean ± standard deviation $\left(\bar{x}\pm s\right)$, and group comparisons were made by using 2 independent samples *t*-tests. Non-normal distribution of quantitative data was expressed as median (range), and group comparisons were made by using the Mann–Whitney *U* test. *P* < .05 was considered statistically significant.

## 3. Results

### 3.1. Clinical and laboratory indicators

In the group of 10 patients with CSE-DVT, there were 5 males and 5 females, with a gender ratio of 1:1, and the median age of onset was 3.2 years (range: 0.2–12.5 years). The underlying conditions included 4 cases (40%) of acute viral encephalitis (of which 2 were caused by herpes simplex virus), 3 cases (30%) of acute encephalopathy, 1 case (10%) of febrile infection-related epilepsy syndrome, 1 case (10%) of fever-related epilepsy syndrome, and 1 case (10%) of cerebral infarction with intracerebral hemorrhage. In the group of 31 patients with nonconvulsive DVT, there were 20 males and 11 females, with a gender ratio of 1.8:1, and the median age of onset was 2.0 years (range: 0.1–13.0 years). The underlying conditions included 10 cases (32.3%) of severe pneumonia, 7 cases (22.6%) of acute lymphoblastic leukemia in maintenance therapy, 6 cases (19.4%) of sepsis, 3 cases (9.7%) of premature infants, 2 cases (6.4%) of massive gastrointestinal hemorrhage, 1 case (3.2%) of severe β-thalassemia with hematopoietic stem cell transplantation, 1 case (3.2%) of congenital myocardial hypertrophy with heart failure, and 1 case (3.2%) of congenital pulmonary arteriovenous malformation during perioperative period. When clinical findings of thrombosis were observed, all the 10 patients with CSE-DVT (100%) had edema of the affected limb, 5 (50%) had markedly elevated skin temperature, 4 (40%) had pain of the affected limb, and 3 (30%) had cyanosis of the affected limb. All the 31 patients with nonconvulsive DVT (100%) had edema of the affected limb, 16 (51.6%) had markedly elevated skin temperature, 14 (45.2%) had cyanosis of the affected limb, and 11 (34.5%) had pain in the affected limb. Among the laboratory indicators, 7 out of 10 patients with CSE-DVT (70%) had platelet counts (PLT) >300 × 10^9^/L (reference value: 100–300 × 10^9^/L); all the 10 patients had activated partial thrombin time (APTT) within the normal range (25.7–39.0 seconds); all the 10 patients had elevated D-dimer, with 5 (50%) >2.00 mg/L (normal range: 0–0.55 mg/L); 4 (40%) had fibrinogen (FIB) <2.00 g/L (normal range: 2.00–4.00 g/L); and 3 (30%) had antithrombin-III (AT-III) activity <85% (normal range: 85.0%–135.0%). Among the nonconvulsive DVT group, 14 out of 31 patients (45.2%) had PLT > 300 × 10^9^/L; 2 out of 31 (6.5%) had APTT prolonged >10 seconds; 23 out of 31 (74.2%) had elevated D-dimer, with 13 out of 31 (41.9%) >2.00mg/L; 14 out of 31 (45.2%) had FIB < 2.00 g/L; and 16 out of 31 (51.6%) had AT-III < 85%. In the CSE-DVT group, the number of cases with a duration of invasive ventilation support time >72 hours, and the number of cases with infection (including primary infection and hospital acquired infection) and intravenous nutritional support was greater than that in the group of patients with nonconvulsive DVT; the PLT and AT-III levels were higher than those in the nonconvulsive DVT group, while the APTT was lower than that in the nonconvulsive DVT group. The differences were statistically significant (*P* < .05), as shown in Table 1.

**Table 1:** Comparison of clinical and laboratory indicators between children with CSE-DVT and nonconvulsive DVT.

	CSE-DVT (n = 10)	Nonconvulsive DVT (n = 31)	*X*^2^/*Z*	*P*-value
Male [n (%)]	5 (50.0)	20 (64.5)	0.670	.413
Age [median (range), yr]	3.2 (0.2–12.5)	2.0 (0.1–13.0)	−0.471	.638
PICU > 7 days [n (%)]	9 (90.0)	26 (81.0)	0.227	.633
Invasive ventilation support >72 h [n (%)]	7 (70.0)	9 (29.0)	5.333	.021
PICC [n (%)]	7 (70.0)	23 (74.2)	0.068	.795
Infection [n (%)]	9 (90.0)	16 (51.6)	4.682	.030
Intravenous nutrition [n (%)]	6 (60.0)	6 (25.0)	6.034	.014
Glucocorticoid use [n (%)]	7 (70.0)	12 (19.0)	2.977	.084
Edema of the affected limb [n (%)]	10 (100)	31 (100)	–	–
Cyanosis of the affected limb [n (%)]	3 (30.0)	14 (45.2)	0.716	.397
Pain of the affected limb [n (%)]	4 (40.0)	11 (34.5)	0.066	.797
Elevated skin temperature of the affected limb [n (%)]	5 (50.0)	16 (51.6)	0.008	.929
WBC [$\left(\bar{x}\pm s\right)$, ×10^9^/L]	12.29 ± 5.08	7.88 ± 6.07	2.070	.045
PLT [$\left(\bar{x}\pm s\right)$, ×10^9^/L]	427 ± 249	262 ± 161	2.448	.019
Hb [median (range), g/L]	90 (74–112)	89 (65–176)	−0.030	.976
Albumin [$\left(\bar{x}\pm s\right)$, ×10^9^/L]	35.95 ± 3.43	34.64 ± 5.63	0.691	.494
APTT [$\left(\bar{x}\pm s\right)$, s]	22.62 ± 4.67	31.24 ± 5.40	−4.419	.000
FDP [median (range), mg/L]	5.30 (4.80–29.30)	7.70 (0.70–43.20)	−0.114	.909
D-dimer [median (range), mg/L]	1.45 (0.94–6.36)	1.11 (0.16–10.54)	−0.934	.350
FIB [median (range), g/L]	1.70 (1.21–3.15)	2.01 (0.65–5.68)	−1.345	.179
AT-III [$\left(\bar{x}\pm s\right)$, %]	111.79 ± 37.41	79.47 ± 25.47	2.673	.012

APTT = activated partial thromboplastin time, AT-III = antithrombin III, CSE = convulsive status epilepticus, DVT = deep vein thrombosis, FDP = fibrinogen degradation products, FIB = fibrinogen, Hb = hemoglobin, PICC = peripherally inserted central catheter, PICU = pediatric intensive care unit, PLT = platelet counts, WBC = white blood cell.

In the CSE-DVT group, the deep veins of the lower extremities were all involved on one side, and 8 cases (80%) involved 2 or more deep veins. In the nonconvulsive DVT group, 15 cases (48.3%) involved the deep vein of the lower extremity unilaterally, 6 cases (19.4%) involved the deep vein of the upper extremity unilaterally, 4 cases (12.9%) involved the subclavian vein, 3 cases (9.7%) involved the hepatic portal vein, 2 cases (6.5%) involved the internal jugular vein, and 1 case (3.2%) involved the inferior vena cava. Among them, 17 cases (54.8%) involved 2 or more deep veins, as shown in Table 2.

**Table 2:** CSE combined with DVT and nonconvulsive DVT children’s thrombosis site overview.

Deep vein site	CSE-DVT (n = 10)	Nonconvulsive DVT (n = 31)
Common iliac vein		
Left	3	2
Right	1	1
External iliac vein		
Left	3	5
Right	2	5
Common femoral vein		
Left	4	6
Right	3	5
Deep femoral vein		
Left	1	0
Right	2	2
Popliteal vein		
Left	1	1
Right	0	1
Subclavian vein		
Left	0	1
Right	0	3
Axillary vein		
Left	0	1
Right	0	2
Brachial vein		
Left	0	1
Right	0	2
Internal jugular vein		
Left	0	1
Right	0	1
Hepatic portal vein	0	3
Inferior vena cava	0	1

CSE = convulsive status epilepticus, DVT = deep vein thrombosis.

### 3.2. Diagnosis, treatment, and prognosis

All patients with DVT were started on anticoagulation therapy immediately after diagnosis. In the group with CSE-DVT, 6 out of 8 patients (75%) received low-molecular-weight heparin (LMWH) combined with aspirin, 2 out of 8 patients (25%) received aspirin alone, 1 out of 8 patients (12.5%) received sequential use of LMWH, warfarin combined with aspirin, and 1 out of 8 patients (12.5%) received warfarin combined with aspirin. In the nonconvulsive DVT group, 19 out of 31 patients (61.3%) received LMWH combined with aspirin, 4 out of 31 patients (12.9%) received aspirin alone, 3 out of 31 patients (9.7%) received sequential use of LMWH, warfarin combined with aspirin, and 2 out of 31 patients (6.4%) received warfarin combined with aspirin. Three out of 31 patients (9.7%) of neonatal portal vein thrombosis patients did not receive anticoagulants. The average follow-up was 6 ± 2 months. In the CSE-DVT group, 5 out of 10 patients (50%) had thrombus absorption, 5 out of 10 patients (50%) had old thrombus formation, all of the patients did not have pulmonary embolism (PE), and no death occurred in either of the 2 groups. In the nonconvulsive DVT group, 16 out of 31 patients (51.6%) had thrombus absorption, 15 out of 31 patients (48.4%) had old thrombus formation, 2 out of 31 patients (6.4%) had PE, and none of the patients died, as shown in Table 3.

**Table 3:** Comparison of treatment and prognosis of CSE-DVT versus nonconvulsive DVT in children.

	CSE-DVT (n = 10)	Nonconvulsive DVT (n = 31)	*X*^2^/*Z*	*P*-value
LMWH + Aspirin [n (%)]	6 (60.0)	19 (61.3)	0.005	.942
LMWH + Warfarin + Aspirin [n (%)]	1 (10.0)	3 (9.7)	0.001	.976
Warfarin + Aspirin [n (%)]	1 (10.0)	2 (6.4)	0.140	.708
Aspirin [n (%)]	2 (20.0)	4 (12.9)	0.305	.581
Non-anticoagulated [n (%)]	0 (0.0)	3 (9.7)	1.044	.307
Thrombus resolution [n (%)]	5 (50.0)	16 (51.6)	0.008	.929
Old thrombosis [n (%)]	5 (50.0)	15 (48.4)	0.008	.929
Pulmonary embolism [n (%)]	0 (0.0)	2 (6.4)	0.678	.410
Thrombus resolution time [median (range), d]	30 (7–60)	24.5 (5–60)	−0.465	.642

LMWH: 100–200 IU/kg d × 5 to 7 days; Aspirin: 3 to 5 mg/kg d; Warfarin: 0.05–0.12 mg/kg d.

LMWH = low molecular weight heparin.

## 4. Discussion

CSE is one of the most common critical conditions in childhood, and with the progress of understanding the causes and treatment methods, its mortality rate is significantly lower than that of adults. Meanwhile, relevant studies have also found that CSE patients often have cognitive regression, motor dysfunction, etc. But unfortunately, current studies pay little attention to their non-neurological complications.^[2]^ This study investigates the clinical features and prognosis of children with CSE-DVT for the first time and found that these patients are mostly affected by deep veins in one leg, involving 2 or more deep veins, with edema, pain, cyanosis, and increased skin temperature being the main clinical features. D-dimer is significantly elevated, with 50% of the patients having D-dimer >2.00 mg/L. Overall, the prognosis is good, with 50% of the patients having thrombus absorption after standard anticoagulation treatment. Compared with the 3.2% incidence rate of DVT combined with PE in adults,^[9]^ no PE cases were found in children with CSE-DVT in this study.

Venous thromboembolism (VTE), which includes DVT and PE, occurs in children at a rate of approximately 0.0007% to 0.0014%,^[2]^ but in hospitalized children, the incidence can reach up to 0.58%.^[10]^ Its occurrence is related to the body’s hypercoagulable state, blood stasis, and vascular endothelial damage. In children, the most common trigger is the central venous access device (CVAD), with approximately 90% of neonates and 60% of older children having VTE related to it.^[11,12]^ In this study, 70% of children with CSE and DVT had a history of CVAD, which is consistent with previous research results. It reminds us to strictly adhere to the indications for CVAD in children with CSE and to properly monitor CVAD devices to prevent DVT.

The management of VTE in children, with limited prior experience, is often based on adult standards, but children are not miniature adults and have unique pathophysiological characteristics, so individualized management is also needed based on evidence-based medicine for the management of VTE in children.^[7]^ Once diagnosed, there are 2 mainstays of treatment: anticoagulation with continued monitoring or thrombolysis.^[13]^ The optimal dosage for systemic thrombolytic therapy of VTE in children remains unclear. Therefore, routine thrombolytic therapy for VTE in children is not recommended, unless there is severe organ or limb damage or hemodynamic instability caused by major vascular occlusion. If thrombolytic therapy is necessary, tissue plasminogen activator or recombinant human tissue plasminogen activator should be used instead of other thrombolytic agents.^[14]^ LMWH is the preferred choice for initial anticoagulant therapy. Compared with unfractionated heparin, LMWH has a higher bioavailability, a longer half-life, and a more predictable anticoagulant effect. Generally, laboratory monitoring is not required during the clinical use of LMWH.^[15]^ Monitoring anticoagulant therapy to achieve outcomes within the target range in childhood VTE, parenteral administration of medications, and frequent blood tests in children are often cumbersome. The availability of safe and effective oral agents with pediatric data to support use would be of clear benefit. Therefore, non-vitamin K oral anticoagulants, including direct thrombin inhibitors (dabigatran) and factor Xa inhibitors (rivaroxaban, apixaban, and edoxaban), are also among the commonly used anticoagulants. Among them, dabigatran and rivaroxaban have been the most thoroughly studied in pediatric patients. Countries such as the United States, Canada, and those in Europe have approved these 2 medications for the treatment and secondary prevention of VTE in pediatric patients.^[16,17]^ Based on EINSTEIN-Jr clinical trial data, rivaroxaban was approved to treat VTE and prevent its recurrence in children of all ages.^[18]^ Other studies have confirmed that rivaroxaban was well tolerated, effective, and safe.^[19,20]^ However further real-world data and observational studies are essential to investigate the use of rivaroxaban among different risk groups. Therefore, for children with asymptomatic DVT or PE, an individualized choice of anticoagulation or no anticoagulation is needed. Anticoagulation therapy is a necessary treatment option for children with symptomatic DVT or PE, and routine thrombolysis, interventional thrombectomy, or implantation of an inferior vena cava filter is not recommended. However, for children with abnormal hemodynamics in PE, it is recommended to start thrombolytic therapy sequentially followed by anticoagulation treatment because these children are often in critical condition and anticoagulation alone may not be effective, requiring rapid thrombolytic therapy to restore normal hemodynamics.^[7]^ For children whose standard anticoagulation treatment is ineffective, anticoagulant thromboplastin replacement therapy can be cautiously selected, but the indications should be strictly selected, such as children with hereditary thromboplastin deficiency, acute lymphoblastic leukemia induced by asparaginase use, nephrotic syndrome, after liver transplantation, and disseminated intravascular coagulation or neonates.^[7]^ CVAD is the most common cause of DVT in children; for children with DVT and normal use of CVAD, it is not necessary to routinely remove it, and simple anticoagulation treatment is sufficient. However, if symptoms persist despite standard anticoagulation treatment, it is still recommended to remove the CVAD. For CVAD that is blocked or nonfunctional, anticoagulation treatment should be given for 3 to 5 days before removal.^[7,11,21]^ For some special types of thrombi, such as portal vein thrombosis, if it is a nonobstructive thrombus or has already formed portal hypertension, anticoagulation treatment may not be needed, and the condition should be monitored while monitoring the changes, and anticoagulation treatment can be given as needed.^[7,22]^ In this study, all 3 infants with portal vein thrombosis who did not receive anticoagulation treatment were able to absorb the thrombus within 1 month, achieving good therapeutic effects. Therefore, the anticoagulant therapy is summarized as follows. It is recommended to use anticoagulant drugs for children with symptomatic DVT. For the initial acute-phase treatment of children newly diagnosed with VTE, it is advisable to administer parenteral anticoagulant therapy with LMWH instead of unfractionated heparin for at least 5 days. For subsequent treatment (after the initial 5–10 days): for children aged 12 years and above, rivaroxaban is recommended for maintenance therapy; for patients aged between 2 and 12 years, a non-vitamin K oral anticoagulants with sufficient evidence-based support or LMWH can be selected; for patients under 2 years of age, LMWH is recommended instead of other anticoagulant drugs.^[23]^

This study also found that approximately 50% of children with CSE-DVT who received only anticoagulant therapy had residual thrombi. Therefore, whether DVT children can be thrombolyzed during the acute phase also deserves further discussion. Currently, there is still controversy over thrombolytic therapy, and the mainstream approach is still to take standard anticoagulation as the main means of treatment and individualized adjustments for different children.^[24,25]^ With the continuous advancement of research, some scholars believe that thrombolytic therapy for DVT children can reduce the recurrence rate of thrombi,^[26,27]^ and local single-root deep vein thrombolysis is better than systemic thrombolysis.^[26]^ However, the evidence is not sufficient, and there is also a risk of bleeding at the thrombolytic site. Some studies have reported a microbleeding incidence of up to 50%.^[28]^

However, the understanding of CSE-DVT is not sufficient, and there have been no relevant reports. In this research, several parameters were observed to differ significantly between the CSE combined with the DVT group and the nonconvulsive DVT group. In the CSE-DVT group, patients required invasive ventilator support for over 72 hours, with a higher number of infection cases, greater utilization of intravenous nutritional support, elevated levels of PLT and AT-III, and a lower APTT. The disparity was statistically meaningful. The invasive ventilator support time is an indicator of the severity of a child’s illness. Firstly, the employment of sedation and analgesia measures leads to extended periods of bed rest, reducing muscle contraction activity and, consequently, insufficient extrusion of venous blood in the lower extremities. Secondly, during the operation of the ventilator, the positive pressure mode often exerts a certain pressure on the large veins in the thoracic cavity, posing resistance to the return of blood from these veins and potentially impeding the return of blood from the deep veins of the lower limbs, and all the aforementioned factors may result in slow and stagnant blood flow.^[29]^ Collectively, these factors contribute to sluggish and stagnant blood flow. Moreover, intravenous nutritional support frequently necessitates central venous cannulation (CVAD). This procedure can cause damage to vascular endothelial cells, thereby augmenting the risk of DVT. In severe infections, the body releases copious amounts of inflammatory mediators and adhesion molecules, which damage the vascular endothelium, exposing subendothelial collagen. This exposure activates platelets and the coagulation system. Simultaneously, the inhibitors of FIB activator increase and are released in large quantities,^[30]^ preventing the efficient and timely removal of fibrin. Instead, fibrin is deposited in microvascular beds, leading to the formation of microthrombi. These mechanisms culminate in a hypercoagulable state of the blood.

Therefore, it is evident that early seizure control and a reduction in ventilator use in children with CSE can further shorten the duration of bed rest and intensive care unit stay. Additionally, early enteral nutrition and proactive infection control can mitigate the incidence of DVT. In summary, based on the current situation, we still need to pay more attention to the basic and clinical research of children with DVT, to provide a definite and beneficial scheme and evidence-based medicine for the good prognosis of these children.

### 4.1. Limitations

First, this is a retrospective study with a small sample size, which precludes multifactor analysis. Consequently, its stability and universality are limited. Second, owing to its retrospective nature, selection bias may be present. Moreover, as a small-sample retrospective study, the limited sample size restricts its generalizability to other regions and ethnic groups. There are numerous etiologies of status epilepticus, and potential confounding factors may exist, potentially leading to analytical errors. Subsequently, we plan to initiate a multicenter prospective study to further explore relevant issues.

## Author contributions

**Conceptualization:** Chao Tang.

**Data curation:** Jie Ren, Chao Tang.

**Formal analysis:** Jie Ren, Chao Tang.

**Funding acquisition:** Jie Ren, Chao Tang.

**Investigation:** Jie Ren, Chao Tang.

**Methodology:** Dan Sun, Jie Ren, Chao Tang.

**Project administration:** Dan Sun.

**Resources:** Jie Ren, Chao Tang.

**Software:** Jie Ren, Chao Tang.

**Supervision:** Chao Tang.

**Validation:** Dan Sun, Chao Tang.

**Visualization:** Dan Sun, Chao Tang.

**Writing – original draft:** Jie Ren, Chao Tang.

**Writing – review & editing:** Dan Sun.
